# Cholesterol biosynthesis pathway as a novel mechanism of resistance to estrogen deprivation in estrogen receptor-positive breast cancer

**DOI:** 10.1186/s13058-016-0713-5

**Published:** 2016-06-01

**Authors:** Nikiana Simigdala, Qiong Gao, Sunil Pancholi, Hanne Roberg-Larsen, Marketa Zvelebil, Ricardo Ribas, Elizabeth Folkerd, Andrew Thompson, Amandeep Bhamra, Mitch Dowsett, Lesley-Ann Martin

**Affiliations:** The Breast Cancer Now Toby Robins Research Centre, The Institute of Cancer Research, London, SW3 6JB UK; Department of Chemistry, University of Oslo, 0371 Oslo, Norway; Academic Department of Biochemistry, Royal Marsden Hospital, London, SW3 6JJ UK; Proteomics Core Facility, Institute of Cancer Research, London, SW3 6JB UK

**Keywords:** Breast cancer, Estrogen receptor, Cholesterol biosynthesis, Oxysterol, Transcriptomics, Proteomics

## Abstract

**Background:**

Therapies targeting estrogenic stimulation in estrogen receptor-positive (ER+) breast cancer (BC) reduce mortality, but resistance remains a major clinical problem. Molecular studies have shown few high-frequency mutations to be associated with endocrine resistance. In contrast, expression profiling of primary ER+ BC samples has identified several promising signatures/networks for targeting.

**Methods:**

To identify common adaptive mechanisms associated with resistance to aromatase inhibitors (AIs), we assessed changes in global gene expression during adaptation to long-term estrogen deprivation (LTED) in a panel of ER+ BC cell lines cultured in 2D on plastic (MCF7, T47D, HCC1428, SUM44 and ZR75.1) or in 3D on collagen (MCF7) to model the stromal compartment. Furthermore, dimethyl labelling followed by LC-MS/MS was used to assess global changes in protein abundance. The role of target genes/proteins on proliferation, ER-mediated transcription and recruitment of ER to target gene promoters was analysed.

**Results:**

The cholesterol biosynthesis pathway was the common upregulated pathway in the ER+ LTED but not the ER– LTED cell lines, suggesting a potential mechanism dependent on continued ER expression. Targeting the individual genes of the cholesterol biosynthesis pathway with siRNAs caused a 30–50 % drop in proliferation. Further analysis showed increased expression of 25-hydroxycholesterol (HC) in the MCF7 LTED cells. Exogenous 25-HC or 27-HC increased ER-mediated transcription and expression of the endogenous estrogen-regulated gene *TFF1* in ER+ LTED cells but not in the ER– LTED cells. Additionally, recruitment of the ER and CREB-binding protein (CBP) to the *TFF1* and *GREB1* promoters was increased upon treatment with 25-HC and 27-HC. *In-silico* analysis of two independent studies of primary ER+ BC patients treated with neoadjuvant AIs showed that increased expression of *MSMO1*, *EBP*, *LBR* and *SQLE* enzymes, required for cholesterol synthesis and increased in our *in-vitro* models, was significantly associated with poor response to endocrine therapy.

**Conclusion:**

Taken together, these data provide support for the role of cholesterol biosynthesis enzymes and the cholesterol metabolites, 25-HC and 27-HC, in a novel mechanism of resistance to endocrine therapy in ER+ BC that has potential as a therapeutic target.

**Electronic supplementary material:**

The online version of this article (doi:10.1186/s13058-016-0713-5) contains supplementary material, which is available to authorized users.

## Background

Over 80 % of breast cancers (BCs) express estrogen receptor alpha (ER) at primary diagnosis. ER is a transcription factor that controls proliferation and survival by binding and activating estrogen response elements (ERE) on target genes controlling proliferation and survival. Current clinical strategies include the use of endocrine agents, which inhibit estrogen (E) signalling either by blocking the conversion of androgens to E in the case of aromatase inhibitors (AIs), by directly antagonizing ER function with agents such as tamoxifen, which compete with E for the ER and results in the recruitment of nuclear corepressors, or by use of drugs such as fulvestrant (ICI182780), which targets ER for proteasomal degradation [1–4].

Despite the efficacy of endocrine therapies, many patients relapse with either *de-novo* or acquired resistance [4–6]. Preclinical and clinical data support cross-talk between ER and growth factor receptor pathways, such as IGF1R and ERBB2/HER2 [7–10], which can lead to ligand-independent activation of the ER or can alter the phosphorylation state of nuclear co-activators, thereby changing the balance of ER transcription factors and potentiating transcription [11]. Despite this knowledge, few clinical trials have shown benefit from the targeting of endocrine resistance using signal transduction or receptor tyrosine kinase inhibitors. One explanation for this is the complexity/heterogeneity of the tumour background and the lack of definitive biomarkers. Data from large studies such as The Cancer Genome Atlas (TCGA) indicate that other than a small number of high-frequency mutations, such as *TP53*, *PIK3CA* and *GATA3*, which have little association with endocrine resistance [12], primary ER+ BC shows a very low frequency of individual mutations, making targeting difficult. In contrast, expression profiling of primary ER+ BC samples has identified several promising signatures/networks for targeting [13].

Previously, using global gene expression data from patients treated with neoadjuvant anastrozole, we showed that certain gene signatures such as IGF-1, MAPK and obesity were associated with poor response to therapy [13]. In order to identify common adaptive pathways associated with acquired resistance to AIs, we derived a panel of cell lines modelling resistance to E-deprivation and analysed these using both high-throughput transcriptomic and proteomic data. The cholesterol biosynthesis pathway was identified as a common adaptive mechanism only in models that retained ER at the point of resistance. Of particular note, the oxysterols 25-hydroxycholesterol (HC) and 27-HC were shown to influence ER transcriptional activity via recruitment to endogenous E-regulated genes. Furthermore, *in-silico* interrogation of data from two separate patient cohorts treated with neoadjuvant AIs or adjuvant tamoxifen showed that genes identified within our *in-vitro* models encoding enzymes within the cholesterol biosynthesis pathway were associated with poor outcome. Overall, these data provide further links between obesity and BC risk.

## Materials and methods

### Cell culture

All wild-type (wt) cell lines (MCF7, HCC1428, SUM44, T47D, ZR75.1) were cultured in phenol red-free RPMI supplemented with 10 % FBS and 1 nM estradiol (E2). Long-term oestrogen-deprivation (LTED) cell lines were cultured in phenol red-free RPMI in the absence of exogenous E2 and supplemented with 10 % dextran charcoal-stripped bovine serum (DCC) [14]. Samples were harvested at baseline, 1 week post E-deprivation and at the point of resistance (LTED). To model the tumour microenvironment during acquisition of resistance to LTED, wt-MCF7 cells were grown on collagen (3 mg/ml) and were referred to as 3D culture.

### Gene expression analysis

RNA was extracted using RNeasy columns (Qiagen, Crawley, UK), according to the manufacturer’s protocol. RNA amplification, labelling and hybridization were conducted on HumanHT-12_V4 Expression BeadChips (Illumina, San Diego, CA, USA), according to the manufacturer’s instructions. Data were normalized using variance-stabilizing transformation (VST) and robust spline normalization (RSN) in the Lumi package [15] [GEO Accession number, GSE75971]. Each triangular comparison per cell line was normalized separately. Probes that were not detected in any sample (detection *p* > 0.01) were discarded. Triangular pairwise comparisons were carried out using BRB-ArrayTools [16]. Differentially expressed probes were considered using the following criteria: False Discovery Rate (FDR) < 5 %, unadjusted *p* < 0.001 and absolute fold-change ≥1.5. Pathway analyses were performed using Ingenuity Pathway Analysis (IPA).

### Proteomics

Peptides from wt-MCF7 and MCF7 LTED cells were isotopically labelled directly using Sep-pak C18 cartridges, as described previously [17, 18]. The wt cells were labelled with the medium isotope reagent and the MCF7 LTED cells with the light isotope reagent. After labelling, each sample was eluted using 80 % acetonitrile with 2 % formic acid. Subsequently, the two labelled samples were pooled at an approximate 1:1 ratio and dried down under vacuum. The dried sample was reconstituted in OFFGEL (OGE, Agilent 3100 OFFGEL Fractionator Kit) stock solution and run using 12 cm IPG strip pH 3–10. Fractions were desalted (SUM SS18V; The Nest Group Inc., Southborough, MA, USA) and run through liquid chromatography-tandem mass spectrometry (LC-MS/MS) using LTQ Velos Orbitrap MS. For MS analysis, the data acquisition mode was set to acquire one full-scan spectrum (350–1850 m/z). After a survey scan, the 20 most intense precursor ions were selected for subsequent fragmentation. For collision-induced dissociation, normalized collision energy was set to 35 %, *q* value to 0.25 and activation time to 10 ms. The isolation width was set to 1.5 and the dynamic exclusion to 1. Raw data were processed using MaxQuant 1.5.1.0 following guidelines by the developers [19–21]. Light and medium dimethyl labels (+28.0313 Da and +32.0564 Da, respectively) were searched at lysine residues and peptide N-termini. Resulting peptide and protein lists were filtered to an estimated FDR of 1 % and 5 %, respectively. Search parameters were chosen as follows: carbamidomethylation was set as a fixed modification on all cysteines. Oxidation of methionines and *N*-acetylation were considered variable modifications. Precursor ion mass tolerance was set to 20 ppm for the first search and fragment ion mass tolerance was set to 0.6 Da. The ‘Re-quantify’ and ‘match between runs’ options were enabled. The automatic decoy search option was also enabled. Spectra were searched for a match to fully-tryptic peptides with up to two missed cleavage sites. All proteomics data are deposited with the PRIDE database with the accession number PXD004085.

### Proliferation assays

Cells were seeded in 10 % DCC into 96-well tissue culture plates and allowed to acclimatize overnight. To assess response of the LTED lines in the presence of estrogen, monolayers were treated with 10 % DCC with or without the presence of escalating concentrations of E2. The medium was replaced after 3 days and cells were cultured for a total of 6 days. For the siRNA knockdown studies, cells were seeded in 10 % DCC and transfected with sicontrol (non-targeting pool) or siRNA targeting *MSMO1*, *IDI1*, *SQLE*, and *EBP* (ON-TARGETplus siRNA; GE Dharmacon, Little Chalfont, Buckingshire, UK) using lipofectamine RNAimax (Invitrogen, Grand Island, NY, USA) [22], according to the manufacturer’s protocol. After 24 hours, monolayers were then treated with 10 % DCC with or without the presence of 1 nM E2 and cells cultured for a total of 6 days. Each experiment was performed at least twice with eight replicates per treatment. To assess the effect of oxysterols or cholesterol, wt-MCF7 cells were stripped from E2 for 72 hours. They were then seeded into 96-well plates and were allowed to acclimatize for 24 hours. The medium was changed on day 3. The cells were treated for 6 days. Cell viability was determined using the CellTitre-Glo® Luminescent Cell Viability Assay (Promega, Madison, WI, USA), according to the manufacturer’s protocol. Values were expressed as fold-change relative to the vehicle-treated control. As a secondary analysis, absolute cell number was also assessed using coulter counts, as described previously [23].

### Transcription assays

The wt cells were stripped of E2 as described previously and seeded into 24-well plates [24]. The following day, the cells were transfected with an ERE-linked luciferase reporter (EREtkLuc) and β-galactosidase constructs using Fugene® 6 (Promega, Madison, WI, USA) [24]. The luciferase activity (Promega, Madison, WI, USA) and β-galactosidase (GalactoStar; Applied Biosystems, Paisley, Scotland, UK) were measured using a luminometer. Each experiment was performed in triplicate and the luciferase values were normalized to the β-galactosidase.

### Western blot

Protein extracts were generated as described previously [23]. Equal amounts of protein (25 μg) were resolved by SDS-PAGE and subjected to immunoblot analysis. Antigen–antibody interactions were detected with ECL reagent (Amersham, Amersham, UK). Proteins were detected using the following antibodies: ER (sc-8002; Santa Cruz Biotechnology, Dallas, Texas, USA) and α-tubulin (Sigma-Aldrich, Poole, Dorset, UK). Secondary antibodies were used at concentration of 1/2000 (Dako, Denmark).

### Quantitative RT-PCR

RNA was extracted 24 hours after treatments using RNeasy columns and quantification was performed using a NanoDrop 1000 spectrometer. cDNA was generated using the SuperScript III First Strand Synthesis System for RT-PCR (Invitrogen). cDNA was subjected to quantitative PCR in triplicate (Applied Biosystems, Paisley, Scotland, UK). Taqman gene expression assays (Applied Biosystems, Paisley, Scotland, UK) were used for *TFF1* (Hs00907239_m1) together with *FKBP15* (Hs00391480_m1) as the housekeeping gene, to normalize the data. The relative quantity was determined using ΔΔCt.

### Chromatin immunoprecipitation quantitative PCR

Chromatin immunoprecipitation (ChIP) experiments were performed, as described previously [25]. Cells were synchronized with α-amanitin [26] and then treated for 45 minutes with 27-HC or 25-HC (10 μM) [27] and fixed. The antibodies used were anti-ER (sc-543 X; Santa Cruz Biotechnology, Dallas, Texas, USA), anti-CBP (sc-369 X; Santa Cruz Biotechnology, Dallas, Texas, USA) and mouse IgG1 (Dako). The resulting DNA was subjected to quantitative PCR analysis using SYBR green (Applied Biosystems, Paisley, Scotland, UK) with the following primers: for *TFF1*, forward 5′-GGC CAT CTC TCA CTA TGA ATC ACT TCT GCA-3′ and reverse 5′-GGC AGG CTC TGT TTG CTT AAA GAG CGT TAG-3′; and for *GREB1*, forward 5′-GAA GGG CAG AGC TGA TAA CG-3′ and reverse 5′-GAC CCA GTT GCC ACA CTT TT-3′.

### Estradiol assay

E2 was measured in whole cell extracts and in cell culture media from the target cell lines using a radioimmunoassay with minor modifications. The intra-assay and inter-assay coefficient of variation (CV) for E2 at a mean of 32 pmol/l was 12 % and 20 %, respectively [28].

### Oxysterol measurements

Cells were grown under basic conditions up to 70–80 % confluency, harvested and cell numbers counted using a Beckman coulter counter. Cells were then pelleted and resuspended in 300 μl ice-cold ultra-pure ethanol (Rathburn Chemicals Ltd, Walkerburn, Peeblesshire, UK) containing 400 nM cholesterol-25,26,27-^13^C_3_. Oxysterols were quantified as described previously [29].

### *In-silico* modelling analysis of 25-HC and 27-HC

Docking of 27-HC and 25-HC was carried out using SwissDock [30, 31] to obtain docking orientations. The most favourable orientation in the ER ligand-binding domain (LBD) was selected. Slight manual manipulation was performed to obtain a better correlation between the binding of E2 and 27-HC or 25-HC within the binding pocket, as well as similar hydrogen bonding.

### Clinical analysis

The clinical relevance of the observations made in this study was tested in two clinical cohorts of patients with primary ER+ BC treated with neoadjuvant anastrozole [32, 33] or letrozole [34]. In the first study, response was based on the Ki67 value less than 10 % after 2 weeks of therapy [12, 35]. In the second study, a reduction in tumour volume of ≥50 % was defined as response [34]. *p* values were calculated using the Mann–Whitney *U* test with *p* < 0.05 regarded as significant. Spearman’s rank correlation was used for the associations between on-treatment gene expression and 2-week Ki67 protein expression. All p values reported are two tailed.

To test the impact in patients treated with tamoxifen, we generated an integrated BC cohort of 747 unique baseline sample profiles based on four publicly available gene expression datasets (GSE6532, GSE9195, GSE17705 and GSE26971). All samples were from patients with ER+, HER2–, tamoxifen-treated (followed by AIs in some cases) disease, without chemotherapy and with follow-up information of relapse-free survival (RFS) up to 10 years. The raw data were normalized on an Affymetrix platform basis: HG-U133A; or HG-U133plus2 using just the MAS function from the simpleaffy R package (http://www.bioconductor.org/) to a mean target intensity of 600, without background correction. The two normalized datasets were then merged and the batch effects across the cohorts were corrected using the ComBat from Surrogate Variable Analysis (sva) R package. Significant impact on RFS was assumed with log-rank *p* < 0.05. The samples were stratified into two groups by the median of gene expression.

The same parameters were used to evaluate the cholesterol biosynthesis genes on 496 ER– patients treated with chemotherapy, excluding endocrine therapy [36]. A significant impact on RFS was assumed to be present with log-rank *p* < 0.05.

## Results

### Transcriptomic and proteomic analysis showed upregulation of cholesterol biosynthesis pathway was restricted to ER+ LTED cells

**Fig. 1:**
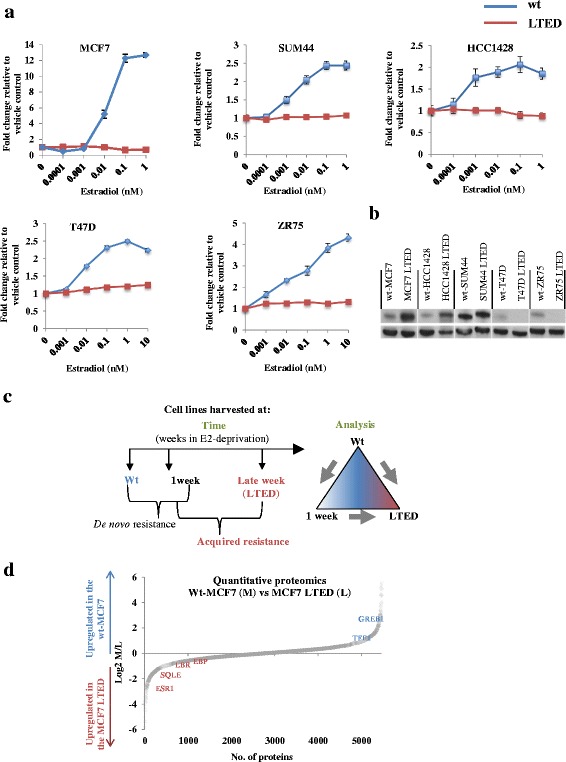
LTED cell lines are refractory to the addition of exogenous E2. **a** wt and LTED cell lines were treated for 6 days with escalating concentration of E2. Proliferation was expressed as the fold-change relative to vehicle-treated control. Data shown are representative of three independent biological experiments with eight replicates per treatment. *Bars*: ± SEM. **b** Western blot analysis assessing ER expression in wt cell lines versus their LTED derivatives. T47D and ZR75.1 lost ER expression, whilst MCF7, SUM44 and HCC1428 retained ER expression. **c** Schematic representation of the triangular pairwise comparison of this study and a diagram depicting the temporal harvesting of samples during acquisition of resistance. **d** Protein profile graph depicting differentially abundant proteins between wt-MCF7 and the MCF7 LTED using dimethyl labelling. *E2* estradiol, *LTED*, long-term estrogen deprivation, *wt* wild type

To identify novel mechanisms of resistance to E-deprivation, we first generated five LTED cell lines. wt-MCF7, wt-ZR75.1, wt-T47D, wt-HCC1428 and wt-SUM44 cells were cultured in the absence of E2 until their growth rate was shown to be independent of the exogenous E2 (Fig. 1a). At the point of resistance, ZR75.1 LTED and T47D LTED cells lost expression of ER whilst MCF7 LTED, SUM44 LTED and HCC1428 LTED cells retained or even upregulated ER expression (Fig. 1b). Pellets derived from the wt cells (representing primary diagnosis) 1-week post E2-deprivation (representing clinical response to AI therapy) and LTED (modelling relapse on an AI) were harvested and global gene expression was interrogated for each cell line using a triangular pairwise comparison (FDR < 5 %, univariate *p* < 0.001 and absolute fold-change ≥1.5) (Fig. 1c). Varying numbers of genes were altered at each time point and differed among cell types, indicating heterogeneity in response to E2-deprivation (Additional file 1: Table S1). As noted previously, comparison between gene changes in wt cells and those deprived of E2 for 1 week were dominated by proliferation, as was the comparison between 1-week deprivation and LTED [24]. We therefore restricted our pathway analysis to comparison of the wt cell lines with their corresponding LTED derivative, in order to remove the confounding effect of proliferation. To identify common adaptive pathways, the IPA software was employed. Strikingly, the cholesterol biosynthesis pathway (Additional file 2: Figure S1) [37–40] was upregulated exclusively in all LTED derivatives that retained expression of ER both in 2D and 3D culture (Table 1). Further interrogation of the gene expression data showed that genes encoding enzymes within the cholesterol biosynthesis pathway were increased significantly (Additional file 3: Table S2).

**Table 1:**
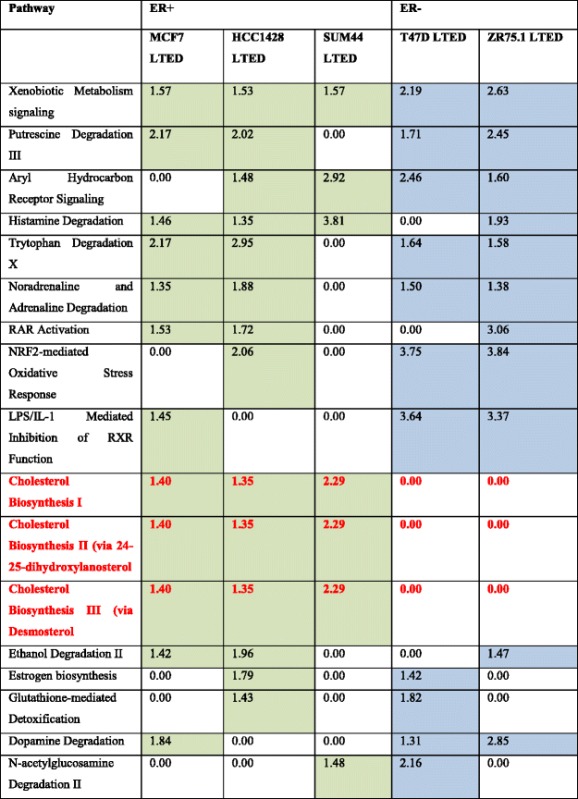
Cholesterol biosynthesis pathways were exclusively upregulated in LTED cell lines retaining ER+

Numbers represent –log_10_ *p* values. Green data depict ER+ cell lines. Blue data depict ER– cell lines

*ER* estrogen receptor alpha, *LTED* long-term estrogen deprivation

As protein and mRNA expression is often disconnected, we carried out quantitative proteomic analysis of wt-MCF7 and MCF7 LTED cells using chemical labelling [17, 18]. Approximately 5000 proteins were identified and 1009 were upregulated in the MCF7 LTED cells compared with wt-MCF7 (Fig. 1d) using the following filter: FDR ≤ 5 % and absolute fold-change ≥1.5. Within the proteins identified were ESR1, in keeping with our western blot analysis, together with GREB1, TFF1 and GATA3 (Additional file 4: Table S3). Pathway analysis confirmed the upregulation of the cholesterol biosynthesis pathway in the MCF7 LTED cells (Additional file 5: Table S4). In particular, enzymes such as sterol 8-isomerase (EBP) (fold-change wt/LTED = –1.54), Lamin-B receptor (LBR) (fold-change wt/LTED = –1.86) and squalene epoxidase (SQLE) (fold-change wt/LTED = –3.2) were evident (Fig. 1d).

### Cholesteryl esters are not associated with the LTED phenotype

Cholesterol plays a major role in both metabolism and cellular architecture. Recent studies have provided a role for cholesteryl esters fuelling the mechanism of invasion and migration and have been associated with more aggressive tumour phenotypes. In particular, recent evidence has provided a link between accumulation of cholesteryl esters and loss of *PTEN* or hyperactivation of the PI3K/AKT/mTOR axis [41, 42]. Upregulation of the PI3K pathway has been associated with resistance to E2-deprivation [43], so we hypothesized that accumulation of cholesteryl esters may be associated with the LTED phenotype. However, assessment of the gene and protein expression of acetyl-CoA acetyltransferase (*ACAT1*), 3-hydroxy-3-methylglutaryl-CoA reductase (*HMGCR*), sterol regulatory element-binding protein (*SREBP1*) and low-density lipoprotein receptor (*LDLR*) [41, 44] – central mediators of this pathway – showed that they were either undetected or downregulated (Additional file 6: Tables S5 and Additional file 7: Tables S6).

### 25-HC and 27-HC enhance ER-mediated transcription in the ER+ LTED models

We hypothesized that the upregulation of the cholesterol biosynthesis pathway may be related to E2 signalling, since cholesterol is the master precursor of the sterol synthesis cascade. To address this, we measured the intracellular as well as the secreted E2 levels in the LTED cell lines; however, in both cases E2 levels were less than 3pmol. Furthermore, exogenous cholesterol showed no impact on ER-mediated transcription in our LTED models (data not shown).

**Fig. 2:**
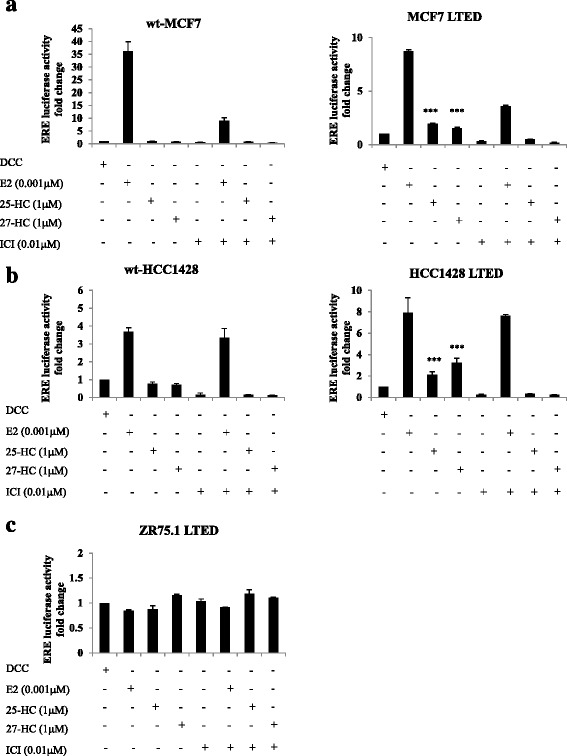
25-HC and 27-HC increase ER transactivation in LTED cell lines retaining ER. Cell lines were transfected with an ERE-luciferase (EREII-tk-luc) reporter gene and treated with E2, 25-HC and 27-HC alone or in combination with the selective E downregulator fulvestrant (ICI 182780 (*ICI*)). **a** wt-MCF7 and MCF7 LTED. **b** wt-HCC1428 and HCC1428 LTED. **c** ZR75.1 LTED. Data are representative of three replicates for each treatment. *Bars*: ± SEM. *DCC* Dextran-coated charcoal, *ERE* estrogen response elements, *HC* hydroxycholesterol, *LTED*, long-term estrogen deprivation, *wt* wild type

Recent findings have suggested that cholesterol metabolites, such as 27-HC, can act as endogenous ligands for ER [45–47]. In order to investigate this further, we assessed the effect of 25-HC and 27-HC on ER-mediated transcription. wt-MCF7 and MCF7 LTED cells were transfected with an EREtkLuc linked reporter construct and treated with E2, 25-HC and 27-HC alone or in combination with fulvestrant (ICI182780). Both oxysterols increased ER-mediated transcription 2-fold (*p* < 0.002) in the MCF7 LTED but had no effect in wt-MCF7. Furthermore, addition of the pure anti-estrogen fulvestrant, which leads to degradation of ER, suppressed oxysterol-driven ER transactivation (Fig. 2a). These data were confirmed in a separate cell line model, HCC1428 LTED (Fig. 2b), but were not evident in ZR75.1 LTED, which had lost expression of ER (Fig. 2c). Taken together, these data suggest that oxysterols can bind and activate ER.

### 25-HC and 27-HC show selective estrogen receptor modulator activity and can rescue the anti-proliferative effects of fulvestrant

**Fig. 3:**
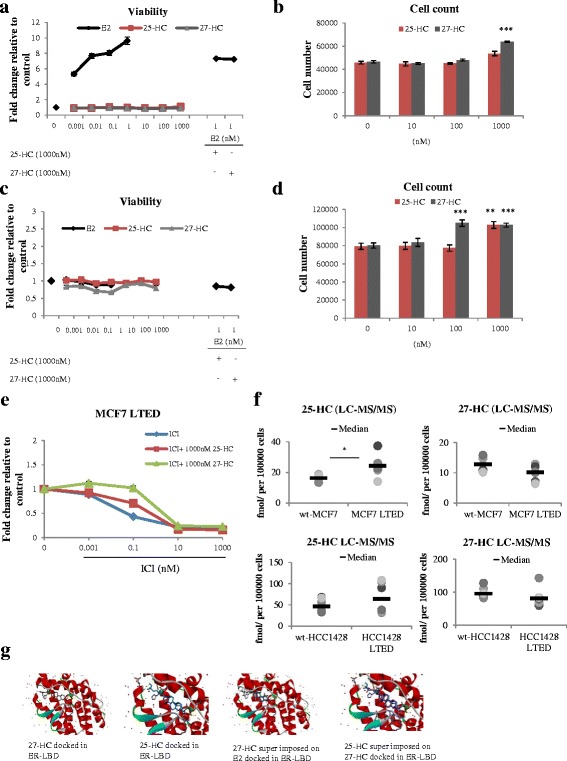
25-HC and 27-HC show SERM activity and can rescue the anti-proliferative effect of fulvestrant. wt-MCF7 and MCF7 LTED cells were treated with increasing (Log_10_M) concentrations of E2, 25-HC or 27-HC alone or in combination for 6 days. Proliferation was measured using TitreGlo to assess changes in cell viability and expressed as fold-change versus vehicle-treated control **a, c** or by assessment of changes in absolute cell number **b, d**. Data shown are representative of eight replicates per treatment for viability and three replicates per treatment for absolute cell number. *Bars*: ± SEM. **e** MCF7 LTED cells were treated with increasing concentrations of fulvestrant (*ICI*) alone or in combination with 25-HC or 27-HC (1000 nM). Cell viability was measured using TitreGlo and expressed as fold-change relative to vehicle-treated control. Data shown are representative of eight replicates per treatment. *Bars*: ± SEM. **f** Levels of 25-HC and 27-HC in whole cell extracts from wt-MCF7, MCF7 LTED, wt-HCC1428 and HCC1428 LTED were measured using LC-MS/MS. Data shown represent the median concentration (fmol/100,000 cells) from two experiments with three biological replicates per cell line. **g** In-silico analysis showing docking of both 27-HC and 25-HC to the LBD of ER based on the crystal structure. *E2* estradiol, *ER* estrogen receptor alpha, *HC* hydroxycholesterol, *LBD* ligand-binding domain, *LC-MS/MS* liquid chromatography-tandem mass spectrometry, *LTED*, long-term estrogen deprivation, *wt* wild type

To assess the effect of 25-HC and 27-HC on the proliferation of both wt-MCF7 and MCF7 LTED, cells were treated with escalating (Log_10_M) concentrations of E2 or the oxysterols. As expected, E2 caused a concentration-dependent increase in proliferation of wt-MCF7 cells but neither 25-HC nor 27-HC showed any significant impact on proliferation when cell viability was measured as the output criterion. In contrast, assessment of absolute cell number showed a small but significant increase (1.4-fold; *p* = 0.0001) in response to 27-HC (Fig. 3b). Of note, addition of 27-HC and 25-HC to E2 showed an antagonistic effect, suggesting that the oxysterols had weak selective estrogen receptor modulator (SERM) activity (Fig. 3a), an observation supported by previous findings [27, 45, 48]. Despite the effect on transcription, proliferation of MCF7 LTED cells was unchanged by addition of E2, 25-HC or 27-HC when measured using cell viability (Fig. 3c). In contrast, assessment of changes in absolute cell number revealed a small but significant increase in response to 25-HC and 27-HC (1.3-fold, *p* = 0.01 and *p* = 0.003, respectively) (Fig. 3d). To investigate this further, we assessed the ability of both 25-HC and 27-HC to rescue the anti-proliferative effect of fulvestrant in the MCF7 LTED (Fig. 3e). As shown previously [23], fulvestrant caused a concentration-dependent decrease in proliferation with an IC_50_ of 0.1 nM. The addition of either of the oxysterols shifted the fulvestrant dose–response curve to the left approximately 10-fold (approximately IC_50_ = 1 nM), suggesting that the oxysterols effectively rescued proliferation (Fig. 3e).

One explanation for the observed results was that the ER+ LTED cells synthesized increased levels of 25-HC and 27-HC, leading to increased ER activity in response to loss of the E2 proliferative signal. To address this, we measured the intracellular levels of the oxysterols using LC-MS/MS. There was a significant increase in the levels of 25-HC in the MCF7 LTED compared with their corresponding parental cell line (Fig. 3f). Furthermore, using the crystal structure of the Activation Function 2 (AF2) [49] domain of ER, which incorporates the LBD, both 25-HC and 27-HC were successfully docked and were superimposed on E2, supporting this hypothesis (Fig. 3g).

### Treatment with 25-HC or 27-HC increases *TFF1* mRNA expression, as a result of enhanced ER recruitment

**Fig. 4:**
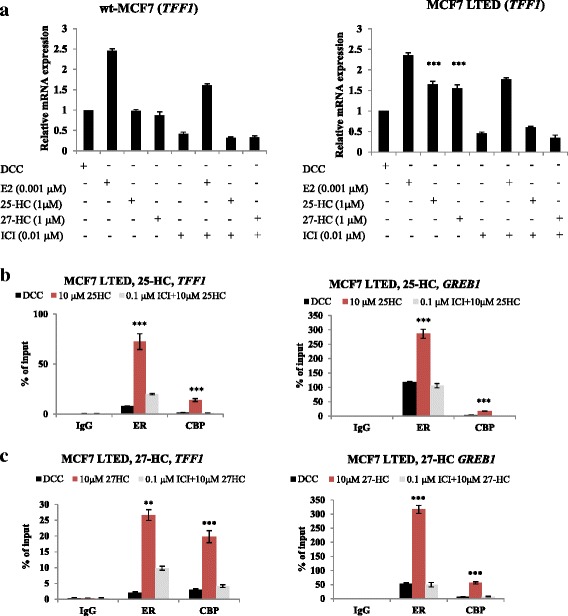
25-HC and 27-HC enhance recruitment of ER to endogenous E-regulated genes in ER+ LTED cells. wt-MCF7 and MCF7 LTED cells were treated with E2, 25-HC and 27-HC alone or in combination with ICI182780 (*ICI*) for 24 hours. mRNA was extracted and quantitative RT-PCR used to measure expression of *TFF1*. **a** Data shown are representative of three independent biological experiments. *Bars*: ± SEM. **b** MCF7 LTED cells were synchronized using α-amanitin and treated with 25-HC for 45 minutes [26]. ChIP was carried out to assess the recruitment of ER to the *GREB1* and *TFF1* promoters, respectively. To provide evidence for an activated complex, histone deacetylase CBP recruitment was also assessed. **c** Effect of 27-HC on ER and CBP recruitment to the *TFF1* and *GREB1* promoters. Data shown are representative of three technical replicates. *Bars*: ± SEM. *DCC* Dextran-coated charcoal, *E2* estradiol, *ER* estrogen receptor alpha, *ERE* estrogen response elements, *HC* hydroxycholesterol, *LTED*, long-term estrogen deprivation, *wt* wild type

To assess this hypothesis further, we measured the effect of exogenous 25-HC and 27-HC on the expression of *TFF1*, an endogenous E-regulated gene. *TFF1* was increased in MCF7 LTED cells in response to both oxysterols, whilst having no effect on *TFF1* expression in wt-MCF7 (Fig. 4a). ChIP of ER and its co-activator CBP in response to both 25-HC and 27-HC showed enhanced recruitment to both the *TFF1* and *GREB1* EREs in the MCF7 LTED cells (Fig. 4b, c).

### Enzymes controlling 25-HC and 27-HC synthesis are associated with poor clinical response in ER+ BC patients

**Fig. 5:**
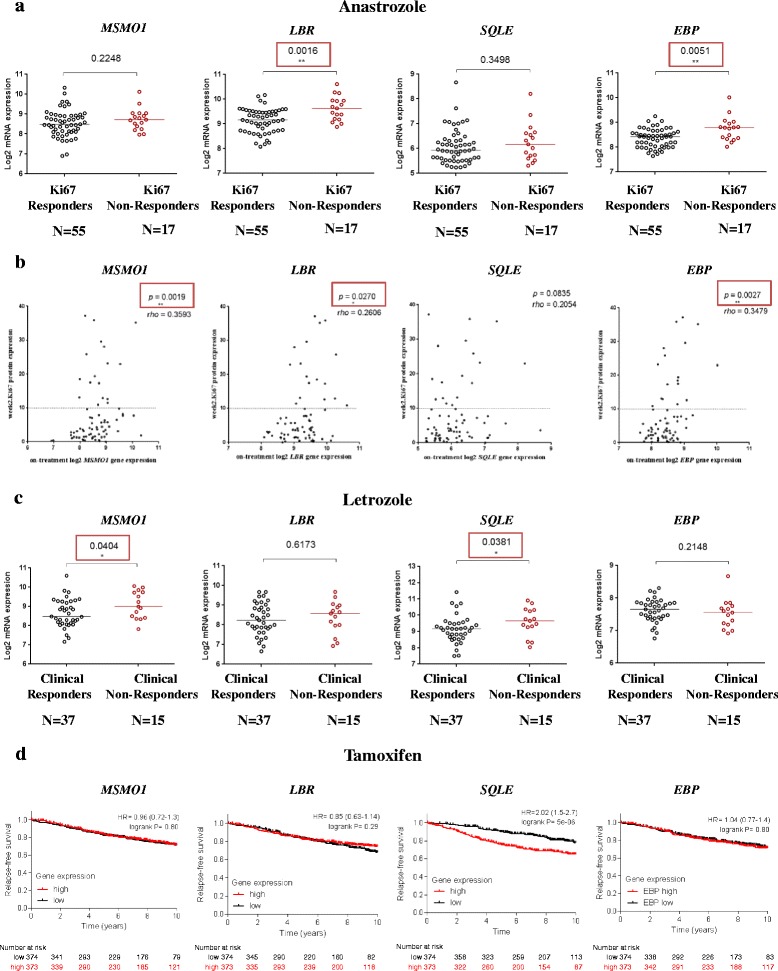
Enzymes within the cholesterol biosynthesis pathway are associated with poor response on tamoxifen. **a** On-treatment gene expression of *MSMO1*, *LBR*, *SQLE* and *EBP* in patients treated with anastrozole. **b** Association of on-treatment gene expression of *MSMO1*, *LBR*, *SQLE* and *EBP* and 2-week Ki67 value. Spearman’s rank correlation coefficients (rho) and *p* values are depicted. **c** On-treatment gene expression of *MSMO1*, *LBR*, *SQLE* and *EBP* in patients treated with letrozole. *Black*, responders; *red*, non-responders. **d** Kaplan–Meier plots revealing the association of high or low pre-treatment gene expression of *MSMO1*, *LBR*, *SQLE* and *EBP* in ER+ BC patients treated with tamoxifen, from publicly available data collected over 10 years. *Red*, patients with higher gene expression; *black*, patients with lower gene expression

To assess the clinical relevance of each of the cholesterol biosynthesis enzymes identified within our transcriptomic and proteomic analysis, we used publicly available datasets for ER+ BC patients treated with neoadjuvant anastrozole [33] or letrozole [34]. Clinical response data were available for 72 patients treated with anastrozole, of which 55 were classed as responders based on a 2-week residual Ki67 score <10 % [12, 35] and 17 were classified as non-responders. In this setting, on-treatment gene expression of *EBP* (*p* = 0.0051) and *LBR* (*p* = 0.0016) were significantly associated with poor response to anastrozole, while the other two candidate genes showed no significant difference (Fig. 5a). We also found that increased on-treatment gene expression of *MSMO1* (*p* = 0.0019), *LBR* (*p* = 0.0270) and *EBP* (*p* = 0.0027) correlated with higher Ki67 expression after 2 weeks of therapy, when expressed as a continuous variable (Fig. 5b). In the second cohort, data were available for 52 tumours, of which 37 were classified as responders on the basis of tumour shrinkage ≥50 %. Increased on-treatment expression of methylsterol monooxygenase 1 (*MSMO1*) (*p* = 0.0404) and *SQLE* (*p* = 0.0381) was strongly associated with poor response to letrozole (Fig. 5c). We next assessed whether expression of these genes was also related to long-term outcome on adjuvant tamoxifen. After 10 years of follow-up of 747 ER+ patients treated with tamoxifen, only *SQLE* (*p* = 5 × 10^–6^) was strongly associated with poor RFS (Fig. 5d). Finally, we assessed the expression of the genes in a cohort of ER– patients treated with chemotherapy and found no significant relationship between expression of the four genes and RFS (Additional file 8: Figure S2A).

### siRNA of individual cholesterol-biosynthesis enzymes has an effect on proliferation

To determine the effect of the enzymes associated with poor RFS (*MSMO1*, *LBR*, *SQLE* and *EBP*) on cell proliferation, we used siRNA knockdown. As expected, inhibition of the four enzymes had no significant effect on ZR75.1 LTED. In contrast, MCF7 LTED showed a 30 % drop in response to si*MSMO1* and si*SQLE* and an approximately 50 % drop in response to siLBR and si*EBP*. Wt-MCF7 showed no significant decrease in response to si*MSMO1*, *LBR* or si*SQLE* but did show an approximately 30 % reduction in proliferation in response to si*EBP*. Of note, the anti-proliferative effect of si*EBP* was not rescued by E2 addition (Additional file 8: Figure S2B).

## Discussion

Despite the success of AIs in treating patients with ER+ BC and reducing their risk of dying from the disease, resistance remains a significant problem [50]. To study novel mechanisms of resistance to E-deprivation on an AI, we generated a panel of cell line models with varying genetic backgrounds and interrogated alterations in both their global gene transcriptome and proteome. Our main focus was to identify common adaptive mechanisms of resistance. Using pathway analysis, we showed a concordant upregulation of the cholesterol biosynthesis pathway in our LTED models that retained ER expression but not in those that lost expression of the steroid receptor.

Evidence suggests both primary and recurrent tumour cells have a high demand of lipids in order to proliferate and metastasize [44]. For instance, activation of the PI3K/AKT/mTOR pathway is strongly associated with lipogenesis [44, 51, 52], as well as accumulation of cholesteryl esters in various cancers via *SREBP1* and *LDLR* activation [41]. In this setting, hyperactivation of PI3K/AKT/mTOR pathway activates SREBP1, thereby potentiating esterification and compartmentalization of cholesterol into lipid droplets, allowing uptake of fatty acids and leading to increased proliferation [42]. As 40 % of BCs harbour activating mutation/amplification of *PIK3CA* or loss of *PTEN*, a feature modelled in our ER+ LTED cell lines, we assessed mRNA levels of *SREBP1*, *LDLR* and *HMGCR*. However, expression at the transcript or protein was either undetected or downregulated. Additionally, *ACAT1*, which is important for cholesterol esterification and prevents cellular toxicity of excess cholesterol, was less abundant in the LTED at both the mRNA and protein level. Furthermore, the levels of esterified cholesterol were not significantly different between wt and LTED cells (data not shown), suggesting this axis is less important in the ER+ LTED phenotype.

There is compelling evidence for the role of cholesterol metabolites in promoting tumour growth [45, 47, 53, 54] and acting as endogenous SERMS [27, 46]. In particular, it has been hypothesized that 27-HC may be the primary biochemical link between lipid metabolism and cancer [55]. We showed that both 25-HC and 27-HC were elevated in our ER+ LTED models compared with their parental controls. Of note, addition of both oxysterols promoted ER-mediated transcription and enhanced recruitment of ER to EREs on both *TFF1* and *GREB1*, two E-regulated genes. Furthermore, this process was antagonized by fulvestrant, suggesting a mechanism dependent on a functional ER-transcription axis. We hypothesized that both 25-HC and 27-HC may substitute for E2 in the LTED setting, a notion supported by our *in-silico* analysis, which showed that both oxysterols were capable of binding with the LBD located in the AF2 domain of ER.

Obesity and lipogenesis have been associated with increased BC risk and a worse outcome as a result of increased levels of adipocyte-secreted endocrine factors and insulin-like growth factors (IGFs) [56, 57]. Previously, we showed that gene signatures modelling both obesity and IGF signalling predicted a poor anti-proliferative effect of anastrozole [13]. To assess whether these enzymes within the cholesterol biosynthesis pathway had relevance for clinical response or prognosis of ER+ BC treated with endocrine therapy that were upregulated in our ER+ LTED models, we interrogated *in-silico* data from patients treated with neoajuvant AI therapy or adjuvant tamoxifen. These analyses revealed that higher expression of four of the nine upregulated genes (*MSMO1*, *EBP*, *LBR* and *SQLE*) correlated with poor response to AI therapy judged either by clinical response [34] or by the validated response biomarker Ki67 [12, 35], but only *SQLE* associated with poor response to endocrine therapy or poor long-term outcome on such therapy or both. Previous studies have shown that *SQLE* is amplified in 10 % of BC [58] and that high expression is associated with poor prognosis in early-stage ER+ BC [59]. Studies have shown that the 8p11-p12 chromosomal region is a hotspot for genomic aberrations and that co-amplification with the *MYC* oncogene (8q24.21) as well as altered transcriptional patterns (hypomethylation) of genes spanning 8q12.1-q24.22, which includes *SQLE*, is associated with more aggressive tumours [60]. Taken together, this would suggest *SQLE* is indicative of poor prognosis while the other cholesterol biosynthesis genes associate more strongly and specifically with poor response to AI therapy. Of note, some genes involved in cholesterol biosynthesis are already incorporated in clinically relevant gene signatures. For instance, DHCR7, which was more abundant in our MCF7 LTED cells (1.25-fold, *p* < 0.001) but did not meet our stringent selection criteria, forms part of the eight-gene EndoPredict signature [61].

## Conclusion

Taken together, these data highlight once again the importance of cholesterol biosynthesis in BC progression and the link with obesity as a marker of increased BC risk and poor outcome on AI therapy [62]. Our observations suggest that enzymes within the cholesterol biosynthesis pathway may be associated with acquired resistance to AI therapy. Clinical measurement, either by gene expression of pertinent enzymes within this complex network or by the assessment of 27-HC and 25-HC, may prove informative biomarkers. Our study highlights the need to evaluate the lowering of cholesterol on the impact of endocrine therapy.

## Additional files

Additional file 1: Table S1. Changes in gene expression during long term estrogen deprivation (LTED). Univariate *p* < 0.001, FDR < 5 %, absolute FC ≥ 1.5. Additional file 2: Figure S1. Schematic representation of the cholesterol biosynthesis pathway (red text indicates enzymes shown to be upregulated in the ER+ LTED cell lines). Additional file 3: Table S2. Alteration in transcript levels for genes within the cholesterol biosynthesis pathway. Additional file 4: Table S3. Validation of proteomic data showing expression of ER and known associated proteins. Protein abundance was measured using dimethyl labelling from wt-MCF7 (M) and MCF7 LTED (L). Changes are represented as fold change (wt-MCF7/MCF7 LTED). Negative values indicate inverted ratios for those < 1. Additional file 5: Table S4. Common upregulated pathways between the proteome and transcriptome in the MCF7 LTED versus wt-MCF7. Values shown as –log p-values. Additional file 6: Table S5. Alteration of *ACAT1* and *LDLR* gene expression in LTED versus their corresponding wt. Additional file 7: Table S6. Comparative abundance of ACAT1, which regulates cholesterol accumulation, presented as fold change (wt-MCF7/MCF7 LTED). Additional file 8: Figure S2.
**A.** Kaplan-Meier plots revealing the influence of high or low expression of *MSMO1*, *LBR*, *SQLE* and *EBP* in relapse-free survival of 496 ER- BC patients treated with chemotherapy, from publicly available data collected over 10 years. **B.** wt-MCF7, MCF7 LTED and ZR75.1 LTED were treated with siRNA targeting siMSMO1, *siLBR*, si*SQLE* and si*EBP*. Wt-MCF7 were treated with or without E2. Change in proliferation was expressed as fold change relative to sicontrol. Bars represent ± SEM from eight replicates. The assessment was carried out in two independent experiments.

## Abbreviations

AI: aromatase inhibitor
BC: breast cancer
ChIP: chromatin immunoprecipitation
DCC: Dextran-coated charcoal
E: estrogen
E2: estradiol
ER: estrogen receptor alpha
ERE: estrogen response elements
HC: hydroxycholesterol
IGF: insulin-like growth factor
IPA: Ingenuity Pathway Analysis
LBD: ligand-binding domain
LC-MS/MS: liquid chromatography-tandem mass spectrometry
LTED: long-term estrogen deprivation
OGE: OFFGEL electrophoresis
RFS: relapse-free survival
SERM: selective estrogen receptor modulator
wt: wild type
