# Supplementary material for: Cholesterol biosynthesis pathway as a novel mechanism of resistance to estrogen deprivation in estrogen receptor-positive breast cancer
Source: Breast Cancer Res. 2016 Jun 1;18:58. doi: 10.1186/s13058-016-0713-5 (PMC4888666; doi:10.1186/s13058-016-0713-5)
Supplement: Additional file 1: Table S1. — Changes in gene expression during long term estrogen deprivation (LTED). Univariate p < 0.001, FDR < 5 %, absolute FC ≥ 1.5. [file 13058_2016_713_MOESM1_ESM.docx]

**Additional file 1. Table S1**

|  | | | | | | |
| --- | --- | --- | --- | --- | --- | --- |
|  | **Wt vs. 1wk deprivation** | | **1wk deprivation vs. LTED** | | **Wt vs. LTED** | |
| **Cell line** | **Up** | **Down** | **Up** | **Down** | **Up** | **Down** |
|  | **(in 1wk)** | **(in 1wk)** | **(in LTED)** | **(in LTED)** | **(in LTED)** | **(in LTED)** |
| **2D MCF7 *** | 1698 | 1512 | 676 | 961 | 512 | 505 |
| **3D MCF7 *** | 485 | 476 | 703 | 780 | 1149 | 1131 |
| **HCC1428 *** | 1246 | 1380 | 218 | 269 | 1181 | 1355 |
| **SUM44 *** | 456 | 431 | 324 | 295 | 191 | 219 |
| **T47D** | 789 | 809 | 939 | 908 | 882 | 912 |
| **ZR75.1** | 214 | 227 | 1205 | 989 | 1129 | 1011 |
|  |  |  |  |  |  |  |
| * LTED phenotype that retains ER+ | | | | |  |  |
