# Supplementary figures and images for: Cholesterol biosynthesis pathway as a novel mechanism of resistance to estrogen deprivation in estrogen receptor-positive breast cancer
Source: Breast Cancer Res. 2016 Jun 1;18:58. doi: 10.1186/s13058-016-0713-5 (PMC4888666; doi:10.1186/s13058-016-0713-5)

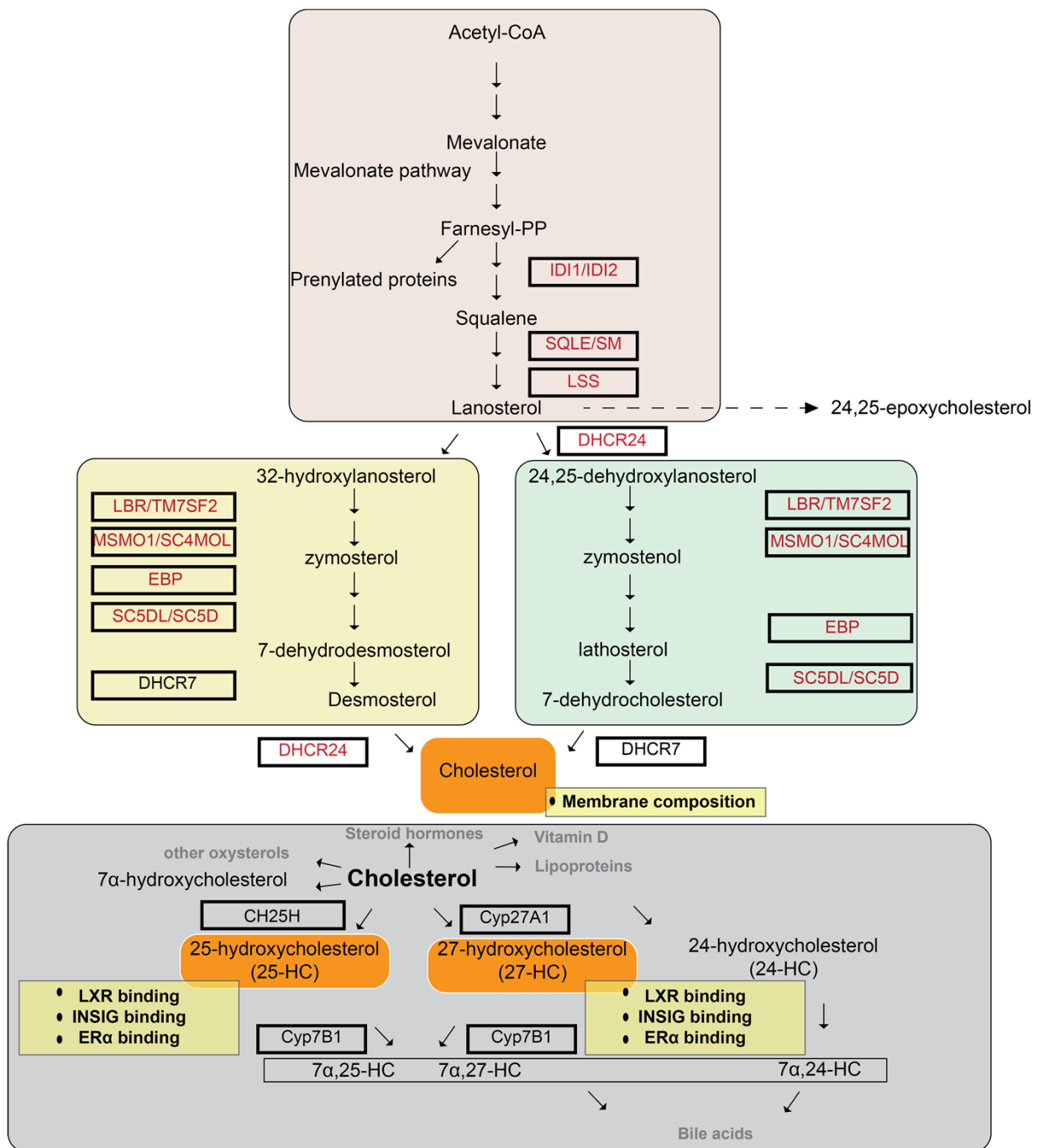

Figure S1

Supplement: Additional file 2: Figure S1. — Schematic representation of the cholesterol biosynthesis pathway (red text indicates enzymes shown to be upregulated in the ER+ LTED cell lines). [file 13058_2016_713_MOESM2_ESM.pdf]

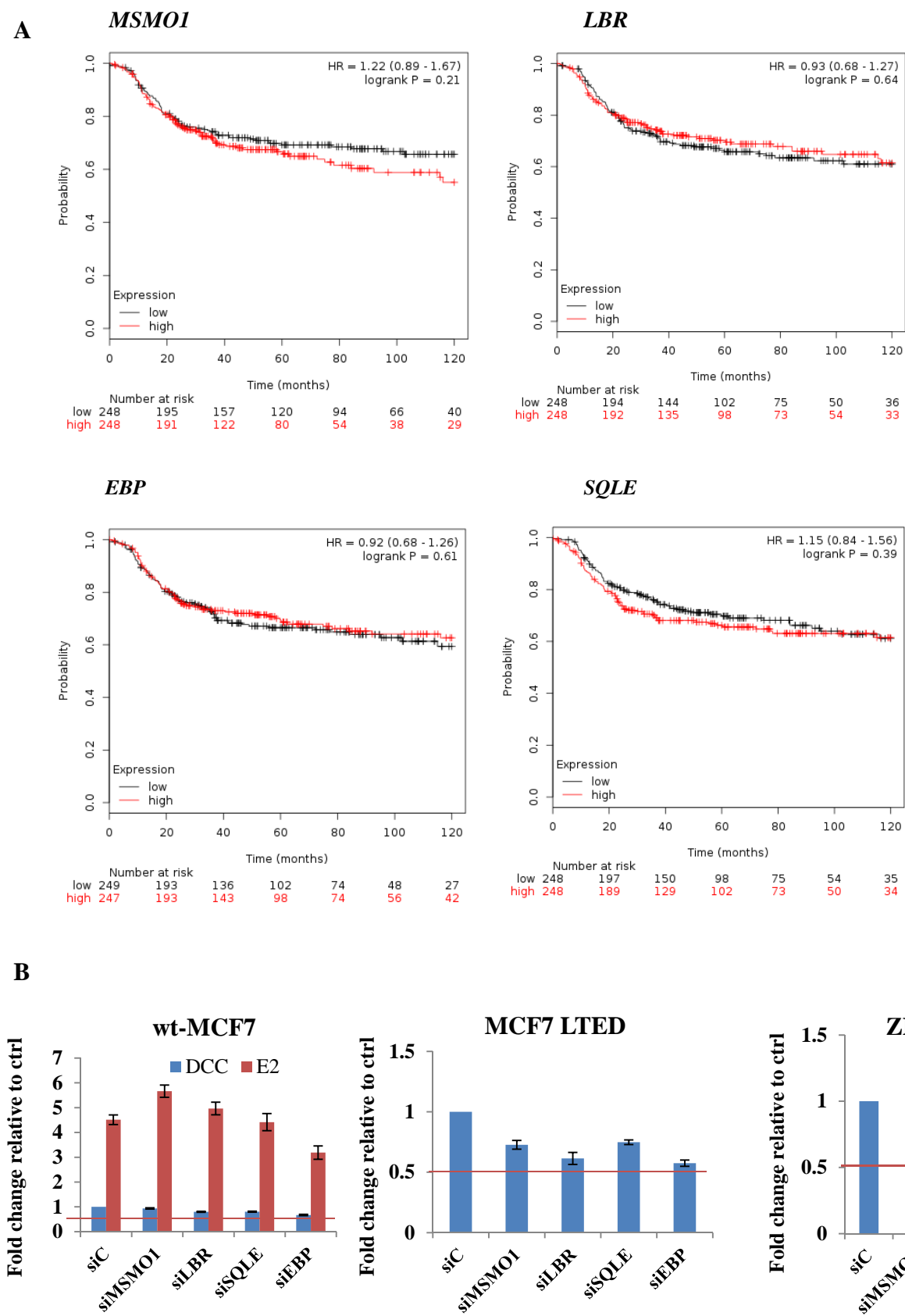

Figure S2

Supplement: Additional file 8: Figure S2. — A. Kaplan-Meier plots revealing the influence of high or low expression of MSMO1, LBR, SQLE and EBP in relapse-free survival of 496 ER- BC patients treated with chemotherapy, from publicly available data collected over 10 years. B. wt-MCF7, MCF7 LTED and ZR75.1 LTED were treated with siRNA targeting siMSMO1, siLBR, siSQLE and siEBP. Wt-MCF7 were treated with or without E2. Change in proliferation was expressed as fold change relative to sicontrol. Bars represent ± SEM from eight replicates. The assessment was carried out in two independent experiments. [file 13058_2016_713_MOESM8_ESM.pdf]
