# Supplementary material for: Cholesterol biosynthesis pathway as a novel mechanism of resistance to estrogen deprivation in estrogen receptor-positive breast cancer
Source: Breast Cancer Res. 2016 Jun 1;18:58. doi: 10.1186/s13058-016-0713-5 (PMC4888666; doi:10.1186/s13058-016-0713-5)
Supplement: Additional file 3: Table S2. — Alteration in transcript levels for genes within the cholesterol biosynthesis pathway. [file 13058_2016_713_MOESM3_ESM.docx]

| **Additional file 3. Table S2** | | | | |
| --- | --- | --- | --- | --- |
| **Gene symbol** | **Cell line (fold change LTED/WT)** | | | |
|  | **MCF7 LTED** **(2D)** | **HCC1428 LTED** | **SUM44 LTED** | **MCF7 LTED (3D)** |
| ***DHCR24*** | 2.01 |  |  | 2.73 |
| ***SC5DL*** |  | 1.86 |  |  |
| ***MSMO1*** |  |  | 1.62 | 2.27 |
| ***LSS*** | 1.63 |  |  |  |
| ***TM7SF2*** |  | 1.54 | 2.59 | 1.6 |
| ***IDI1*** |  |  | 1.95 | 2.74 |
| ***SQLE*** |  |  |  | 2.37 |
| ***EBP*** |  |  |  | 1.57 |
