# Supplementary material for: Cholesterol biosynthesis pathway as a novel mechanism of resistance to estrogen deprivation in estrogen receptor-positive breast cancer
Source: Breast Cancer Res. 2016 Jun 1;18:58. doi: 10.1186/s13058-016-0713-5 (PMC4888666; doi:10.1186/s13058-016-0713-5)
Supplement: Additional file 4: Table S3. — Validation of proteomic data showing expression of ER and known associated proteins. Protein abundance was measured using dimethyl labelling from wt-MCF7 (M) and MCF7 LTED (L). Changes are represented as fold change (wt-MCF7/MCF7 LTED). Negative values indicate inverted ratios for those < 1. [file 13058_2016_713_MOESM4_ESM.docx]

| **Additional file 4. Table S3** | | |
| --- | --- | --- |
| **Gene symbol** | **wt-MCF7/MCF7 LTED (M/L)** |  |
| **ESR1** | -6.75 |  |
| **GATA3** | -2.24 |  |
| **TFF1** | 1.54 |  |
| **GREB1** | 4.28 |  |
