# Supplementary material for: Cholesterol biosynthesis pathway as a novel mechanism of resistance to estrogen deprivation in estrogen receptor-positive breast cancer
Source: Breast Cancer Res. 2016 Jun 1;18:58. doi: 10.1186/s13058-016-0713-5 (PMC4888666; doi:10.1186/s13058-016-0713-5)
Supplement: Additional file 5: Table S4. — Common upregulated pathways between the proteome and transcriptome in the MCF7 LTED versus wt-MCF7. Values shown as –log p-values. [file 13058_2016_713_MOESM5_ESM.docx]

| **Additional file 5. Table S4** | | |
| --- | --- | --- |
| **Pathways (IPA analysis)** | **UP proteome** | **UP transcriptome** |
|  | **MCF7 LTED vs WT** | **MCF7 LTED vs WT** |
| **Cholesterol Biosynthesis I** | **1.69** | **1.4** |
| **Cholesterol Biosynthesis II (via 24,25-dihydrolanosterol)** | **1.69** | **1.4** |
| **Cholesterol Biosynthesis III (via Desmosterol)** | **1.69** | **1.4** |
