# Supplementary material for: Cholesterol biosynthesis pathway as a novel mechanism of resistance to estrogen deprivation in estrogen receptor-positive breast cancer
Source: Breast Cancer Res. 2016 Jun 1;18:58. doi: 10.1186/s13058-016-0713-5 (PMC4888666; doi:10.1186/s13058-016-0713-5)
Supplement: Additional file 6: Table S5. — Alteration of ACAT1 and LDLR gene expression in LTED versus their corresponding wt. [file 13058_2016_713_MOESM6_ESM.docx]

| **Additional file 6. Table S5** | | | | | |
| --- | --- | --- | --- | --- | --- |
| **Gene symbol** | **Cell line (fold change LTED/WT)** | | | | |
|  | MCF7 LTED (2D) | HCC1428 LTED | SUM44 LTED | T47D LTED | ZR75.1 LTED |
| ***ACAT1*** | -1.59 | 0 | 0 | -1.69 | 0 |
| ***LDLR*** | 0 | -1.56 | 0 | 0 | -2.63 |
