# Supplementary material for: Cholesterol biosynthesis pathway as a novel mechanism of resistance to estrogen deprivation in estrogen receptor-positive breast cancer
Source: Breast Cancer Res. 2016 Jun 1;18:58. doi: 10.1186/s13058-016-0713-5 (PMC4888666; doi:10.1186/s13058-016-0713-5)
Supplement: Additional file 7: Table S6. — Comparative abundance of ACAT1, which regulates cholesterol accumulation, presented as fold change (wt-MCF7/MCF7 LTED). [file 13058_2016_713_MOESM7_ESM.docx]

| **Additional file 7. Table S6** | |
| --- | --- |
| **Gene symbol** | **wt-MCF7/MCF7 LTED (M/L)** |
| **ACAT1** | 1.24 |
